# Chemically defined and growth factor-free system for highly efficient endoderm induction of human pluripotent stem cells

**DOI:** 10.1016/j.stemcr.2024.11.012

**Published:** 2024-12-26

**Authors:** Zhiju Zhao, Fanzhu Zeng, Yage Nie, Gang Lu, He Xu, He En, Shanshan Gu, Wai-Yee Chan, Nan Cao, Jia Wang

**Affiliations:** 1 School of Health and Life Sciences, University of Health and Rehabilitation Sciences, Shandong 266071, China; 2 Zhongshan School of Medicine, Sun Yat-Sen University, Guangdong 510080, China; 3 Key Laboratory for Stem Cells and Tissue Engineering (Sun Yat-Sen University), Ministry of Education, Guangdong 510080, China; 4 CUHK-SDU Joint Laboratory on Reproductive Genetics, School of Biomedical Sciences, The Chinese University of Hong Kong, Hong Kong SAR, China; 5 Hong Kong Branch of CAS Center for Excellence in Animal Evolution and Genetics, The Chinese University of Hong Kong, New Territories, Hong Kong SAR 999077, China; 6 Key Laboratory for Regenerative Medicine, Ministry of Education, School of Biomedical Sciences, Faculty of Medicine, The Chinese University of Hong Kong, New Territories, Hong Kong SAR 999077, China; 7 Department of Plastic and Hand Surgery, Klinikum Rechts der Isar, School of Medicine, Technical University of Munich, 81675 Munich, Germany

**Keywords:** definitive endoderm differentiation, high-throughput screening, growth factor-free differentiation system, TEAD3

## Abstract

Definitive endoderm (DE) derived from human pluripotent stem cells (hPSCs) holds great promise for cell-based therapies and drug discovery. However, current DE differentiation methods required undefined components and/or expensive recombinant proteins, limiting their scalable manufacture and clinical use. Homogeneous DE differentiation in defined and recombinant protein-free conditions remains a major challenge. Here, by systematic optimization and high-throughput screening, we report a chemically defined, small-molecule-based defined system that contains only four components (4C), enabling highly efficient and cost-effective DE specification of hPSCs in the absence of recombinant proteins. 4C-induced DE can differentiate into functional hepatocytes, lung epithelium, and pancreatic β cells *in vitro* and multiple DE derivatives *in vivo*. Genomic accessibility analysis reveals that 4C reconfigures chromatin architecture to allow key DE transcription factor binding while identifying TEAD3 as a novel key regulator of the process. This system may facilitate mass production of DE derivatives for drug discovery, disease modeling, and cell therapy.

## Highlights

• A small-molecule-based definitive endoderm (DE) induction system (4C) is developed
• 4C-induced DE differentiates into hepatocytes, lung organoid, and pancreatic β cells
• 4C reconfigures chromatin architecture to allow key DE transcription factor binding
• TEAD3 is a novel key regulator for DE formation

## Graphical abstract

**Figure:**
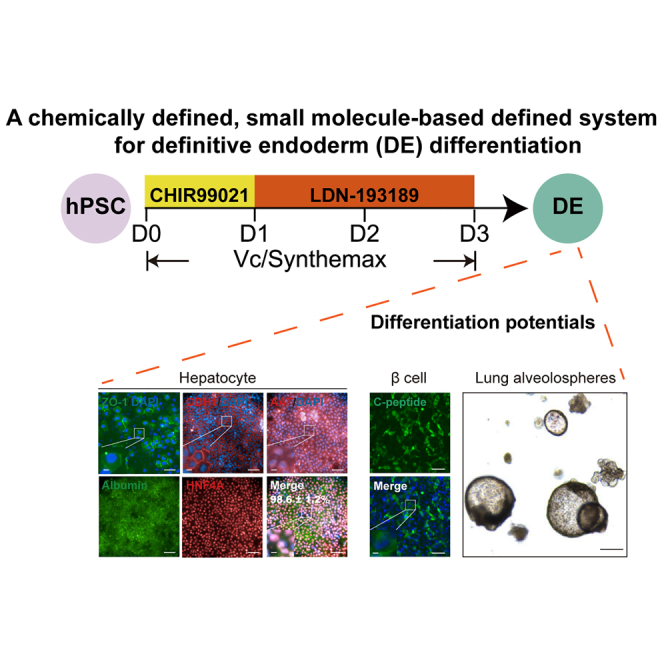

## Teaser

In this article, Wang and colleagues develop a chemically defined system that consists of only four components, enabling highly efficient and cost-effective DE specification of hPSCs in the absence of recombinant proteins. Induced DE can differentiate into functional hepatocytes, lung epithelium, and pancreatic β cells *in vitro*. Mechanical study shows that TEAD3 is a critical regulator for DE formation.

## Introduction

Human pluripotent stem cell (hPSC)-derived definitive endoderm (DE), which can give rise to the respiratory epithelium, hepatocytes, pancreatic cells, and intestinal lineages, holds significant translational value for drug screening, disease modeling, toxicity testing, and cell replacement therapies targeting diseases such as type 1 diabetes and acute liver failure (Cherry and Daley, 2013). Despite numerous protocols established in the past two decades for DE specification of hPSCs including both human embryonic stem cells (hESCs) and human induced pluripotent stem cells (hiPSCs) (Yiangou et al., 2018), achieving homogeneous differentiation remains a major challenge. Moreover, these protocols have traditionally relied on undefined or animal-origin components, such as bovine serum albumin (BSA) and Matrigel (Loh et al., 2014), and/or the inclusion of expensive recombinant human albumin and growth factor of variable potency (Jiang et al., 2021; Korostylev et al., 2017). The presence of complex and undefined components in the medium increases both the cost and batch viability, thereby impeding our understanding of the molecular mechanisms underlying DE specification and hindering industrial-scale cell production that meets “good manufacturing practice” standards for drug testing and screening applications (Yiangou et al., 2018). Furthermore, this poses a significant obstacle to utilizing hPSC-derived DE as therapy products, which require chemically defined and xeno-free conditions.

Most current strategy to direct the differentiation of hPSCs toward a desired cell fate involves mimicking embryonic development, wherein hPSCs are exposed to signaling events that they would typically encounter *in vivo*, such as Activin/Nodal, fibroblast growth factor , bone morphogenetic protein (BMP), and Wnt signaling (Loh et al., 2014). Activin/Nodal signaling pathway plays a determinate role in DE development, which is conserved across *Xenopus laevis*, zebrafish, and the mammalians (Wells and Melton, 1999). Therefore, almost all protocols directing DE differentiation of hPSCs have relied on using recombinant Activin-A (AA) as an indispensable core inducer to date (Bogacheva et al., 2018; Li et al., 2019). The incorporation of high-quality and contamination-free growth factors in DE induction poses one of the biggest challenges for their scalable production and clinical applications. Currently, there is still a lack of a fully defined, recombinant protein-free system that enables highly efficient and cost-effective derivation of DE cells.

To overcome this obstacle, in this study, we have systematically optimized the DE differentiation conditions and have conducted a high-throughput screen to identify chemical substitutes for essential protein components. As a result, we developed a chemically defined system that consists of only four components, enabling highly efficient and cost-effective DE specification of hPSCs in the absence of recombinant proteins.

## Results

### Growth factor-free medium for DE differentiation

We first confirmed the essential role of AA in previously established methods. In the absence of AA, it was observed that H1 hESCs failed to differentiate into any SOX17^+^/FOXA2^+^ DE cells when cultured in undefined conditions containing BSA and Matrigel used by these protocols, even with the presence of CHIR99021, a Wnt signaling activator that serves as a DE inducer (Teo et al., 2014) (Figure S1A). Since hPSCs can produce low levels of endogenous AA/Nodal that support their differentiation (Kattman et al., 2011), it is unclear why hPSCs cannot convert into DE at all in the absence of exogenous AA.

**Figure 1:**
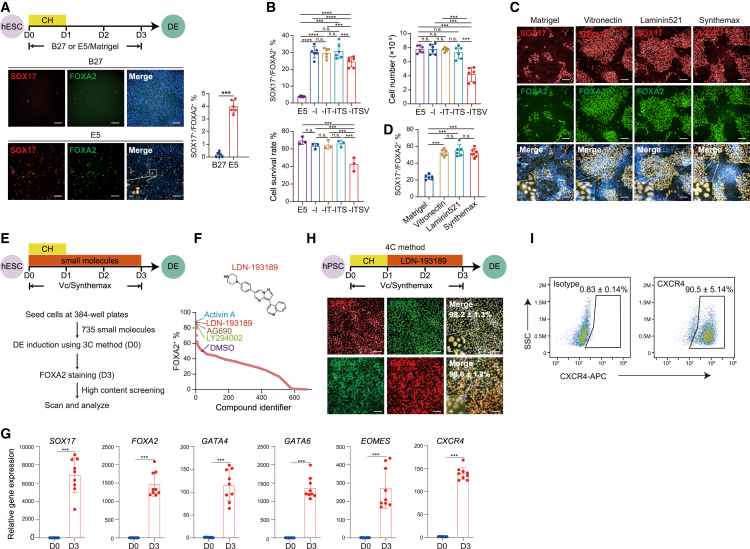
Development of a fully synthetic system for highly efficient DE induction from hPSCs (A) Immunofluorescence analysis of DE markers SOX17 and FOXA2 on hESCs differentiated in E5 or B27 supplement-containing culture medium from 3 days as illustrated by the upper schematic (*n* = 6 biologically independent experiments). CH, CHIR99021. Scale bars, 50 μm. (B) Percentage of SOX17^+^/FOXA2^+^ DE, cell yields, and survival rate of cells under the indicted conditions at D3 (*n* = 6 biologically independent experiments). A minus mark demonstrates withdrawal of the indicated component. I, insulin; T, transferrin; S, sodium selenite; V, vitamin C. (C and D) Representative (C) and quantitative (D) immunofluorescence analysis of SOX17 and FOXA2 on hESCs differentiated on the indicated matrix substrates for 3 days (*n* = 6 biologically independent experiments). Scale bars, 50 μm. (E and F) Workflow (E) and high-throughput small screening results (F) for DE inducers in conjunction with 3C. Activin-A and DMSO serve as positive or negative controls, respectively. Chemical structure of the leading hit LDN-193189 is shown. (G) Quantitative reverse-transcription PCR (RT-qPCR) analyses of the expression of key DE transcripts (*n* = 9 biologically independent experiments). (H) Immunofluorescence analysis of DE markers SOX17, FOXA2, GATA4, and GATA6 with quantification of the SOX17^+^/FOXA2^+^ and GATA4^+^/GATA6^+^ rations on D3 DE induced by 4C illustrated by the upper schematic (*n* = 6 biologically independent experiments). Scale bars, 50 μm. (I) Flow cytometric analyses of the DE marker protein CXCR4 in 4C-DE at D3 (*n* = 6 biologically independent experiments). Data are represented as mean ± SE. ^∗∗∗^*p* < 0.001; ^∗∗∗∗^*p* < 0.0001; n.s., no significant.

We reasoned that the undefined components in BSA and Matrigel, such as the albumin-carried lipids or growth factors typically supporting hPSC self-renewal (Garcia-Gonzalo and Izpisua Belmonte, 2008), could potentially hinder DE formation. To test this hypothesis, we compared published recipes for hPSC culture and differentiation (Chen et al., 2011; Lin et al., 2017; Yasuda et al., 2018) and developed a chemically defined, albumin-free medium formula containing only five ingredients (E5 medium), including the basal medium DMEM/F-12, insulin, transferrin, sodium selenite, and vitamin C (Vc). Notably, application of this chemically defined E5 medium enabled DE specification of H1 hESCs with only CHIR99021, generating ∼4% SOX17^+^/FOXA2^+^ DE cells without the need of exogenous AA supplement (Figure 1A).

To determine if each component in E5 medium was essential, we systematically eliminated individual ingredients and treated the cells with the remaining ones. It was observed that exclusion of insulin significantly enhanced differentiation efficiency to around 30%, as evidenced by immunostaining analyses (Figures 1B and S1B). This finding is consistent with a previous study demonstrating that JNK-JUN signaling, which is downstream of insulin, acts as a critical barrier to DE commitment in hPSCs (Li et al., 2019). Further withdrawal of any component, except Vc, had no detrimental effect on the efficiency of DE induction at differentiation day (D) 3 (Figures 1B and S1B). Moreover, only Vc removal markedly reduced both the yield (Figures 1B and S1B) and survival rate (Figures 1B and S1C) of differentiated cells. Therefore, we have identified a simplified growth factor-free medium consisting solely of Vc and CHIR99021 in DMEM/F-12 as sufficient for DE differentiation of hESCs after removing undefined components.

### A chemically defined and growth factor-free condition for highly efficient and cost-effective DE induction

The growth of hPSCs relies on appropriate extracellular matrix coating, such as the Matrigel utilized in the aforementioned condition. To examine whether replacing the undefined Matrigel with other synthetic or recombinant matrix substrates could further enhance DE specification, we evaluated recombinant vitronectin (Chen et al., 2011) and laminin-521 (Rodin et al., 2014), as well as Synthemax (Jin et al., 2012), a synthetic peptide substrate. We found that each of these defined matrices tested exhibited significantly improved DE induction efficiency (∼50%) compared to Matrigel when used in differentiation medium supplemented with CHIR99021 and Vc (Figures 1C and 1D). For the remainder of this study, we employed Synthemax due to its synthetic nature and relatively cost-effective properties; this condition was designated as 3C (CHIR99021, Vc, and Synthemax).

To identify small molecules that can further enhance DE specification in conjunction with 3C, we systematically screened an in-house-generated chemical library containing 735 epigenetic and signaling pathway modulators. This library was expanded from our earlier study (Wang et al., 2022) and screened in a high-throughput manner, with AA as a positive control. Specifically, the undifferentiated H1 hESCs were seeded into 384-well plates in 3C and a single chemical compound was added to each well by a robotic liquid handling system. Cells were then allowed to differentiate for 3 days followed by immunostaining analyses of FOXA2 expression via a high-content imaging and analyzing system (Figure 1E). We have identified 47 positive hits that increased FOXA2^+^ DE percentage above the DMSO (solvent) control without decreasing cell viability (Table S1). Top hits included LDN-193189, an inhibitor of BMP type I receptors ALK2 and ALK3; AG-690, a poly (ADP-ribose) polymerase-1 inhibitor; and LY294002, a phosphatidylinositol 3-kinase inhibitor. The compounds tested in combination with CHIR99021 and Vc demonstrated robust generation of SOX17^+^/FOXA2^+^ DE cells, achieving comparable efficiency to AA (Figure 1F). After extensive testing, LDN-193189 was identified as the most potent and reproducible DE inducer, following fine-tuning of dose (Figure S1D) and administration duration (Figure S1E). Our protocol successfully validated deterministic induction of DE (up to 98.2% differentiation efficiency revealed by quantifying SOX17^+^/FOXA2^+^ cell percentage at D3) from two hESC lines (H1 and H9) and two hiPSC lines (WTB and WTC) (Figures S1E and S1F). We also compared the DE differentiation efficiency between 4C method and AA-treated method and found that the DE differentiation efficiency is higher using 4C method than AA-treated method (Figure S1F). Thus, we have developed a chemically defined, growth factor/albumin-free system consisting of only four synthetic components (CHIR99021, LDN-193189, Vc, and Synthemax; referred to as 4C hereafter), enabling highly efficient and cost-effective derivation of DE from hPSCs. The cost of implementing the 4C method is estimated to be only approximately 0.6% of that required by AA-based protocols (Table 1).

**Table 1:** Formulation and price of components of medium for AA- or 4C-based method

Medium components	Catalog number	AA method		4C method	
		Concentration	Cost/L (RMB)	Concentration	Cost/L (RMB)
RPMI-1640	Thermo Fisher C11875500BT	1 × 138		–	
DMEM/F12	Thermo Fisher C11330500BT	–		1 × 80	
CHIR99021	TargetMol T2301	3 μM 49		3 μM 49	
Vitamin C	Sigma A8960	–		71 mg L^−1^ 11	
B27 minus ins	Thermo Fisher A3695201	1 × 7796		–	
Activin-A	R&D 338-AC	100 ng mL^−1^ 16688		–	
LDN193189	Selleck S7507	–		0.1 μM 15	
–	–	**Total cost** 24,671/L		**Total cost** 155/L	

### Molecular signature of 4C-induced DE differentiation

To further characterize the 4C-induced DE cells (4C-DE), we assessed the expression of key DE genes. We observed a significant upregulation of many DE-related transcripts at differentiation D3 compared to undifferentiated H1 hESCs (Figure 1G). Additionally, D3 cells exhibited uniform expression of several critical DE proteins, including GATA4, GATA6, and CXCR4, in addition to SOX17 and FOXA2 (Figures 1H and 1I).

**Figure 2:**
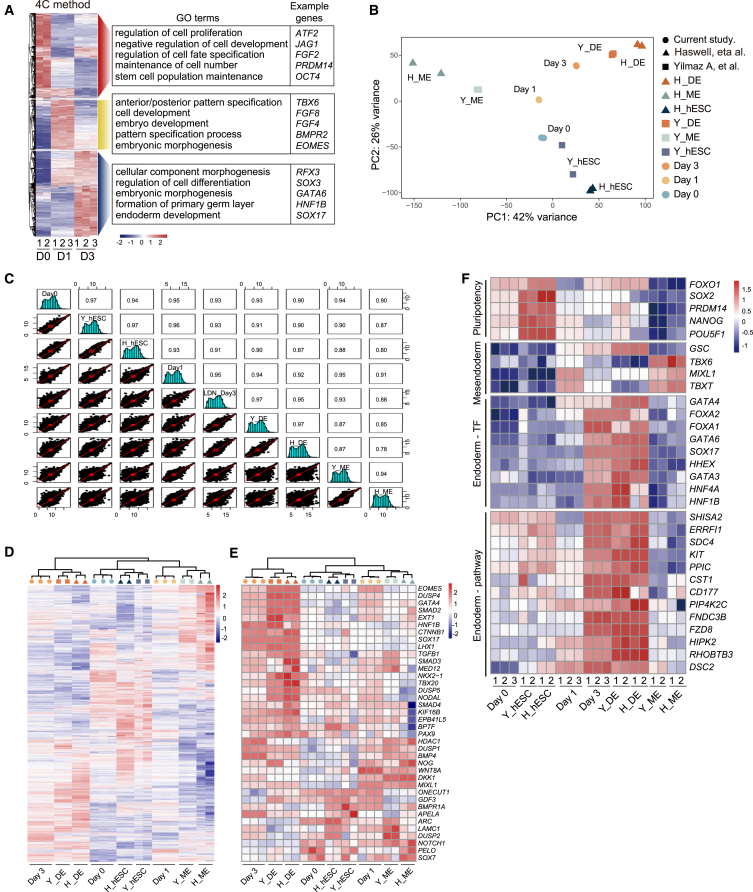
4C-DE exhibits typical DE transcriptional program (A) Heatmap showing differently expressed genes in D0, D1, and D3 cells during 4C-induced DE specification revealed by RNA-seq. Right: the Gene Ontology (GO) analysis of genes in each cluster, along with a representative gene for each GO term. (B) Principal component analysis of the transcriptome across all tested cell types revealed by RNA-seq. H_, Haswell et al.; Y_, Yilmaz et al.; ME, mesoderm. (C) Person’s correlation analysis of the global gene expression profiles across all tested cell types. (D and E) Hierarchical classification analysis of genes that are differently expressed (D) and that fall into the GO term of “endoderm development” (E) among all tested cell types. (F) Expression of pluripotency, mesendoderm, and DE marker genes in all tested samples detected by RNA-seq.

To investigate the transcriptional trajectory of 4C-induced differentiation, we conducted RNA sequencing (RNA-seq) analysis on cell samples at specific time points corresponding to stage-specific transitions in cell state, including pluripotency (D0), mesendoderm (D1), and DE (D3). By hierarchical cluster analysis, we observed a gradual reset of the global transcriptome from D0 to D3 (Figure 2A). In Gene Ontology (GO) analysis, we observed that GO terms enriched in D1 cells were associated with mesendodermal specification, such as “anterior/posterior pattern specification,” whereas genes upregulated in D3 cells were related to terms that are important for DE development, such as “formation of primary germ layer” and “endoderm development”; in contrast, genes downregulated during differentiation were strongly enriched with the gene networks associated with hESC self-renewal and growth (Figure 2A).

To further evaluate the molecular roadmap of 4C-induced differentiation, we compared aforementioned samples with published reference cells at different stages during DE (Haswell et al., 2021) or mesodermal (Yilmaz et al., 2020) differentiation of hESCs. We found that 4C elicited a clear molecular roadmap toward the DE fate (Figure 2B). The transcriptional profile of D3 cells closely resembles that of DE cells generated by other growth factor-based protocols, while being distinct from undifferentiated hESCs or mesodermal cells (Figures 2C–2E). Furthermore, high expression levels of DE-related genes, including transcription factors (TFs) and cell signaling modulators, have been confirmed in D3 cells (Figure 2F). However, they exhibit weak expression of markers associated with pluripotency and unspecified mesendodermal cells (Figure 2F). In aggregate, these data demonstrate that 4C-DE possess key molecular feature characteristic of normal DE cells.

### Differentiation potentials of 4C-DE

**Figure 3:**
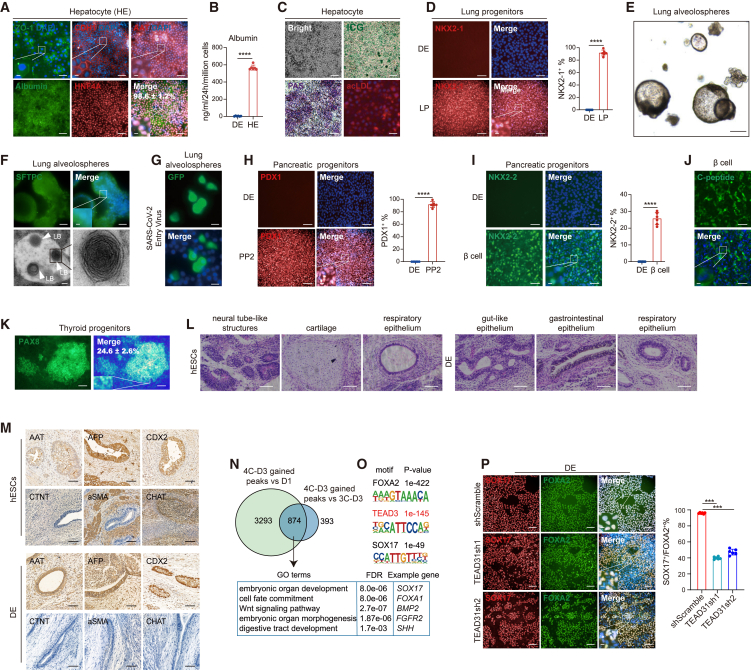
4C-DE is multipotent (A) Immunofluorescence analysis of hepatocyte (HE) markers ZO-1, CHD1, AAT, albumin, and HNF4A in 4C-DE that underwent a 16-day hepatic differentiation. Differentiation efficiency is revealed by quantification of the HNF4A^+^ cell percentage (*n* = 6 biologically independent experiments). Scale bars, 50 μm. (B) ELISA analysis of albumin secretion by 4C-DE-derived hepatocytes at D16 in comparison with the parental 4C-DE (*n* = 6 biologically independent experiments). (C) Cell morphology, indocyanine green (ICG), and acetylated low-density lipoprotein (acLDL) uptake, as well as periodic acid schiff (PAS) staining analysis of 4C-DE-derived hepatocytes at D16. Scale bars, 50 μm. (D) Immunofluorescence analysis of lung progenitor (LP) marker NKX2-1 in 4C-DE or 4C-DE that exposed to the lung AT2 cell differentiation condition for 12 days (*n* = 6 biologically independent experiments). Scale bars, 50 μm. (E) Bright-field microscopy showing the morphology of lung alveolosphere formed at differentiation D22. Scale bars, 25 μm. (F) Immunofluorescence analysis of the lung AT2 cell marker SFTPC (upper, scale bars, 50 μm) and transmission electron microscopy analysis of the formation of lamellar body-like inclusions (lower, scale bars, 0.2 μm) in D22 lung alveolospheres. LB, lamellar body. (G) Fluorescence microscopy examination of GFP expression in lung alveolospheres infected by the SARS-CoV-2 entry virus. Scale bars, 50 μm. (H–J) Immunofluorescence analysis of the stage 2 pancreatic progenitor (PP2) marker PDX1 (H) and pancreatic β cell markers NKX2-2 (I) and C-peptide (J) in 4C-DE or 4C-DE that subjected to pancreatic differentiation condition for 7 (H) and 31 days (I and J), respectively. Scale bars, 50 μm. (K) Immunofluorescence analysis of the thyroid progenitor marker PAX8 in 4C-DE that exposed to the thyroid progenitor differentiation condition for 10 days (*n* = 6 biologically independent experiments). Scale bars, 100 μm. (L and M) Hematoxylin-eosin staining (L) and immunohistochemistry (M) analysis of cellular plugs derived from H1 hESCs or 4C-DE after transplantation into the immunodeficient mice. Scale bars, 100 μm. (N) Venn diagram outlining the overlap between D3 4C-DE-gained peaks compared to D1 or compared to 3C-treated cells at D3. Lower: the GO analysis results of the overlapping genes, each represented by an example gene from its respective GO term. (O) Motif enriched at the overlapping peaks identified in (N). (P) Immunofluorescence analysis of SOX17 and FOXA2 in shScramble control and two TEAD3 knockdown (shTEAD3-1 and shTEAD3-2) hESC lines differentiated with 4C for 3 days (*n* = 6 biologically independent experiments). Scale bars, 50 μm. Data are represented as mean ± SE. ^∗∗∗^*p* < 0.001; ^∗∗∗∗^*p* < 0. 0001; n.s., no significant.

To access the multipotency of 4C-DE, we examined their capacity to differentiate into several known DE derivatives including hepatocytes, lung alveolar epithelial type II (AT2) cells, and pancreatic β cells based on established protocols. Hepatic differentiation was achieved through a stepwise approach (Ang et al., 2018) (Figure S2A), resulting in high expression levels of numerous hepatocyte-specific transcripts (Figure S2B) and proteins, such as ZO-1, CHD1, AAT, albumin, and HNF4A (Figure 3A), as well as secretion of albumin (Figure 3B). The differentiated cells exhibited typical morphological features and functional hallmarks of mature hepatocytes such as glycogen storage capacity, lipid uptake and storage capability, and indocyanine green absorption potential (Figure 3C).

To test the alveolar specification potential of 4C-DE, we exposed them to AT2 differentiation conditions (Jacob et al., 2017) (Figure S2C). We observed highly efficient induction of the NKX2-1^+^ primordia lung progenitors (Figure 3D), which subsequently differentiated into SFTPC^+^ AT2-like cells and formed monolayered epithelial “alveolospheres” in 3D cultures (Figures 3E and 3F). Additionally, we noted that AT2-like cells significantly upregulated numerous alveolar transcripts (Figure S2D). Transmission electron microscopy analysis of these lung organoid-like alveolospheres revealed the formation of lamellar body-like inclusions, a typical feature of lung AT2 cells (Figure 3F). Moreover, we found that these 4C-DE-derived alveolospheres were permissive for severe acute respiratory syndrome coronavirus 2 (SARS-CoV-2) entry as they were infected with a vesicular stomatitis GFP virus pseudotyped with the SARS-CoV-2 spike protein (Ou et al., 2020) (SARS-CoV-2-entry virus) (Figure 3G).

To investigate the pancreatic potential of 4C-DE, we employed a previously published protocol (Shi et al., 2017) (Figure S2E) specifically designed for directing differentiation toward pancreatic β cells and assessed the expression of key markers associated with pancreatic lineage development. We observed significantly upregulation of key genes enriched in pancreatic progenitors and β cells during differentiation (Figure S2F). By immunostaining analyses, we further validated that 4C-DE could generate up to 90% PDX1^+^ pancreatic progenitors (Figure 3H), which gave rise to ∼24% NKX2-2^+^ β cells expressing C-peptide as well (Figures 3I and 3J). Furthermore, we also investigated the thyroid differentiation potential of 4C-DE by employing a well-established method (Kurmann et al., 2015) (Figure S2G). We observed a significant upregulation of many thyroid genes upon differentiation (Figure S2H). Through immunostaining analysis, we discovered that 4C-DE can efficiently differentiate into PAX8^+^ thyroid progenitors with an efficiency of 24.6% (Figure 3K). We then conducted a comparative analysis of the differentiation potential between DE induced by 4C and AA, respectively. Notably, we observed significantly enhanced efficiency in 4C-DE when differentiating into DE derivatives (Figures S2I–S2K). Collectively, these findings suggest that 4C-DE exhibits remarkable multilineage endodermal differentiation potential and efficiently generates functional hepatocytes, lung AT2 cells, pancreatic β cells, and thyroid progenitors *in vitro*.

To further elucidate the *in vivo* differentiation potential of 4C-DE, we implanted them within concentrated Matrigel and subcutaneously transplanted both 4C-DE and undifferentiated hESCs, which served as a control for uncommitted cells, into the groin region of immunodeficient mice. After a period of 9 weeks post transplantation, we examined the cellular plugs derived from transplantation to assess evidence of cellular differentiation. In mice that underwent hESC transplantation, we observed the formation of cells originating from all three germ layers, including ectodermal tissues (e.g., neural tube-like structures), mesodermal tissues (e.g., cartilage), and endodermal tissues (e.g., respiratory epithelium) (Figure 3L). Conversely, transplanted 4C-DE formed various endodermal tissues such as gut-like, gastrointestinal and respiratory epithelium with minimal observation of derivatives from mesoderm or ectoderm (Figure 3L). The immunohistochemistry analysis further confirmed these findings, demonstrating that the transplanted 4C-DE formed tissues exclusively expressing DE derivative-specific genes, including hepatocyte cell markers AAT and AFP, as well as the intestinal epithelial marker CDX2. However, no expression of genes associated with mesoderm lineages such as the cardiomyocyte marker cTNT and smooth muscle marker αSMA was observed, nor was there any expression of ectoderm lineage genes like the neuron marker choline acetyltransferase (Figure 3M). Overall, these *in vivo* data indicate the restricted differentiation potential of 4C-DE to endoderm.

### Chromatin remodeling during 4C-induced DE differentiation

To obtain a more comprehensive understanding of how 4C reconfigures chromatin architecture to specify DE and identify key underlying regulators, we performed assay for transposase-accessible chromatin sequencing (ATAC-seq) on D1, D2, and D3 cells undergoing 4C-induced differentiation as well as on 3C-treated cells (without the DE booster LDN-193189) at D2 and D3 for comparison. Our findings suggest that genomic loci gradually closing from D1 to D3 are typically located near mesoendodermal genes such as *MIXL1*, *TBX6*, and *ID1*. Meanwhile, chromatin states shifting from close to open during the transition from D1 to D3 were frequently associated with genes that promote DE formation including *SOX17*, *GATA6*, and *FOXA2* (Figure S3A). Consistently, when comparing the chromatin accessibility of 3C and 4C-treated cells at D3, we observed that *cis*-regulatory elements showing increased chromatin accessibility in 4C were predominantly located proximal to genes involved in DE specification (Figure S3B). Intersection of these two groups of DE-promoting *cis*-regulatory elements during 4C-induced differentiation identified 874 overlapped accessible chromatin regions that control a total of 769 genes. These genes regulate numerous biological processes related to DE (Figure 3N).

Furthermore, we found that the TF binding motif overrepresented in these open chromatin regions was a sites occupied by many well-characterized regulators of DE, such as FOXA2, GATA6, and SOX17 (Figure 3O). Interestingly, we identified binding sites of TEAD3 as another most preferentially enriched motif in these DE-promoting genomic regions (Figure 3O). Footprinting analysis further confirms TEAD3’s role as a bona fide TF that bounds to these sites (Figure S3C). As an important downstream effector of the Hippo signaling pathway (Ma et al., 2019), TEAD3 expression significantly increases during 4C-induced differentiation (Figure S3D). Given the ambiguity surrounding the involvement of Hippo/TEAD3 in DE development, we employed short hairpin RNAs (shRNAs) to downregulate TEAD3 expression levels (Figure S3E) and assessed its impact on DE differentiation. Our findings suggest that depletion of TEAD3 via shRNAs significantly reduces the population of SOX17^+^/FOXA2^+^ DE cells induced by 4C treatment (Figures 3P and S3F), indicating a crucial role for TEAD3 as a novel regulator of DE formation.

Overall, these data demonstrate that 4C facilitates DE formation by establishing an open chromatin state on regions promoting DE development, thereby allowing key transcription factors to bind and shape the DE transcriptional program.

## Discussion

The use of recombinant proteins in stem cell-based therapies presents significant challenges for cost-effective and efficient culture optimization, as the production of quality-controlled recombinant proteins suitable for clinical applications is expensive. This hinders the cost-effective scale-up of hPSC-derivative production for both clinical and industrial purposes. Small molecules with similar functionality may offer an affordable solution to replace these costly protein components, as they are more stable, convenient to mass manufacture, and cost-effective (Li et al., 2013). Our previous work has demonstrated the efficacy of small molecules in modulating cell fate, which can circumvent the need for foreign genetic materials or recombinant growth factors (Cao et al., 2016; Wang et al., 2022).

In this study, by systematically optimizing the differentiation condition and high-throughput identification of chemical DE inducers, we develop a small-molecule-based defined system that enables deterministic (up to 98.6% efficiency) and cost-effective DE commitment of hPSCs in the absence of albumin and growth factors. The resulting 4C-DE is multipotent and capable of differentiating into cells of liver, lung, pancreas, and thyroid lineages. To the best of our knowledge, the 4C protocol contains fewer components than any other published methods to date and represents the first synthetic ingredient-only system for generating DE (Table S2). It reduces the cost by 99.4% in comparison to the traditional growth factor-based approach and is generally applicable to multiple hESC and hiPSC lines. Due to its simplicity, minimal quality control requirements, low cost, as well as fully chemically defined and xeno-free nature, the 4C system reported here may have significant implications in basic research, drug screening, and translation of DE-related cell therapy into clinical applications.

In conclusion, 4C is a defined, sample, cost-effective, and small-molecule-based defined system for DE differentiation. hPSC-derived endoderm in the 4C system can efficiently differentiate into functional hepatocytes, lung epithelium, and pancreatic β cells, demonstrating their promising potential in drug discovery, disease modeling, and cell therapy. Moreover, TEAD3 is an essential transcription factor for endoderm differentiation in the 4C system.

## Experimental procedures

### Cell lines and culture conditions

Two hESC lines, H1 and H9, and two hiPSC lines, WTB and WTC, were used in this study. H1 (male) and H9 (female) were obtained from WiCell. WTC (male) and WTB were obtained from Conklin lab, Gladstone/UCSF. hESCs and hiPSCs were cultured on Matrigel (Corning, 354277)-, Vitronectin (Thermo, A14700)-, or Synthemax Ⅱ-SC Substrate (Corning, 3535)-coated plates in E8 medium (STEMCELL Technologies, 05940) at 37°C with 5% CO_2_. Cells were passaged every 3–4 days using 0.5 mM EDTA (Thermo Fisher Scientific, AM9260G) in Dulbecco’s phosphate-buffered saline without Ca^2+^ or Mg^2+^ (Gibco, 14190136) at 37°C for 3 min. HEK293T cells (ATCC, CRL-321) were cultured in high-glucose Dulbecco’s modified Eagle’s medium (DMEM, HyClone, SH30022.01) supplemented with 10% fetal bovine serum (HyClone, SH30406.05) and 2 mM GlutaMAX (Gibco, 35050061) at 37°C with 5% CO2. HEK293T cells were passaged with trypsin (Gibco, 25200072) and the culture medium was changed every other day. All cell lines were confirmed to be mycoplasma-free by using the MycoAlert Mycoplasma Detection Kit (Lonza, LT-07-418).

### DE differentiation

AA-based DE differentiation was performed as previously reported (Bogacheva et al., 2018; Garcia-Gonzalo and Izpisúa Belmonte, 2008). Briefly, undifferentiated hESCs cultured in E8 medium were dissociated into single-cell suspension by Accutase (STEMCELL Technologies, 7920) and reseeded onto Matrigel-coated 12-well plate at a density of 2 × 105 cells/per well in E8 medium containing 5 μM Rho kinase inhibitor Y-27632 (Selleck, S1049). When reached ∼80%–90% confluence, DE differentiation was initiated by switching to RPMI1640 medium containing B27 minus insulin supplement (Thermo Fisher Scientific, 175004-44) and cultured for 3 days. 3 μM CHIR99021 (TargetMol, T2301) and 100 ng mL-1 AA (R&D, 338-AC) were added from days 0–1 and days 0–3, respectively.

For 4C-based DE differentiation, undifferentiated hESCs and hiPSCs were similarly dissociated, seeded onto Synthemax-coated plates, and grown to ∼80%–90% confluence. Cells were then cultured in DMEM/F12 medium (Thermo Fisher Scientific, C11330500BT or 11330032) supplemented with 71 μg mL-1 Vc (Sigma, A8960) and cultured for 3 days. 3 μM CHIR99021 and 0.1 μM LDN193189 (Selleck, S7507) were added from days 0–1 and days 2–3, respectively.

See supplemental experimental procedures.

### Quantification and statistical analysis

Values were presented as mean ± SE and quantified from at least three biological repeats unless otherwise stated. Unpaired two-tailed Student’s t test was used for statistical significance between two groups if data are in a normal distribution; otherwise, the Wilcoxon test was used. For comparisons of multiple groups, one-way analysis of variance with a post hoc Tukey test was used. *p* value <0.05 was considered two-sided significant.

The procedures for hepatocytes, lung alveolar cells, pancreatic β cells and thyroid progenitors differentiated from DE, *in vivo* differentiation of DE, RNA-seq, and ATAC-seq are described in the supplemental experimental procedures.

## Resource availability

### Lead contact

Further information and requests for resources and reagents should be directed to and will be fulfilled by the lead contact, Jia Wang (jiawang@uor.edu.cn).

### Materials availability

All the materials generated and used in this study will be available upon reasonable request.

### Data and code availability

Raw and processed RNA-seq and ATAC-seq data have been deposited to NCBI Gene Expression Omnibus (GEO) repository (accession number: GSE274488 for RNA-seq data and accession number: GSE274662 for ATAC-seq data). All other relevant data are available from the corresponding author upon reasonable request.

## Acknowledgments

This work was supported by the 10.13039/501100012166National Key R&D Program of China (2023YFA1801200), the 10.13039/501100001809National Natural Science Foundation of China (92268105, 32430031, 32200685, and 32471160), the 10.13039/501100003453Natural Science Foundation of Guangdong Province (2022A1515011819), the Taishan Scholar Foundation of Shandong Province (tsqn202306271), and the 10.13039/501100010870Qingdao Municipal Science and Technology Bureau (23-2-8-smjk-11-nsh).

## Author contributions

N.C., J.W., and Z.Z. conceived the project and wrote the manuscript. Z.Z. and F.Z. carried out the experiments. Y.N. performed bioinformatic analyses. W.-Y.C. supervised the study, assisted in data analysis, and provided materials. G.L., H.X., H.E., and S.G. performed data analysis, provided materials, edited the manuscript, or assisted with the experiments. All authors contributed to and approved the final manuscript.

## Declaration of interests

N.C., Z.Z., and F.Z. have filed patent applications related to DE differentiation by 4C.

## Supplemental information

Document S1. Figures S1–S3 and Tables S1–S3

Document S2. Article plus supplemental information
