# Supplementary material for: Chemically defined and growth factor-free system for highly efficient endoderm induction of human pluripotent stem cells
Source: Stem Cell Reports. 2024 Dec 26;20(1):102382. doi: 10.1016/j.stemcr.2024.11.012 (PMC11784501; doi:10.1016/j.stemcr.2024.11.012)
Supplement: Document S1. Figures S1–S3 and Tables S1–S3 [file mmc1.pdf]

Stem Cell Reports, Volume *20*

Supplemental Information

Chemically defined and growth factor-free system for highly efficient endoderm induction of human pluripotent stem cells

Zhiju Zhao, Fanzhu Zeng, Yage Nie, Gang Lu, He Xu, He En, Shanshan Gu, Wai-Yee Chan, Nan Cao, and Jia Wang

Fig. S1 (Related to Fig. 1)

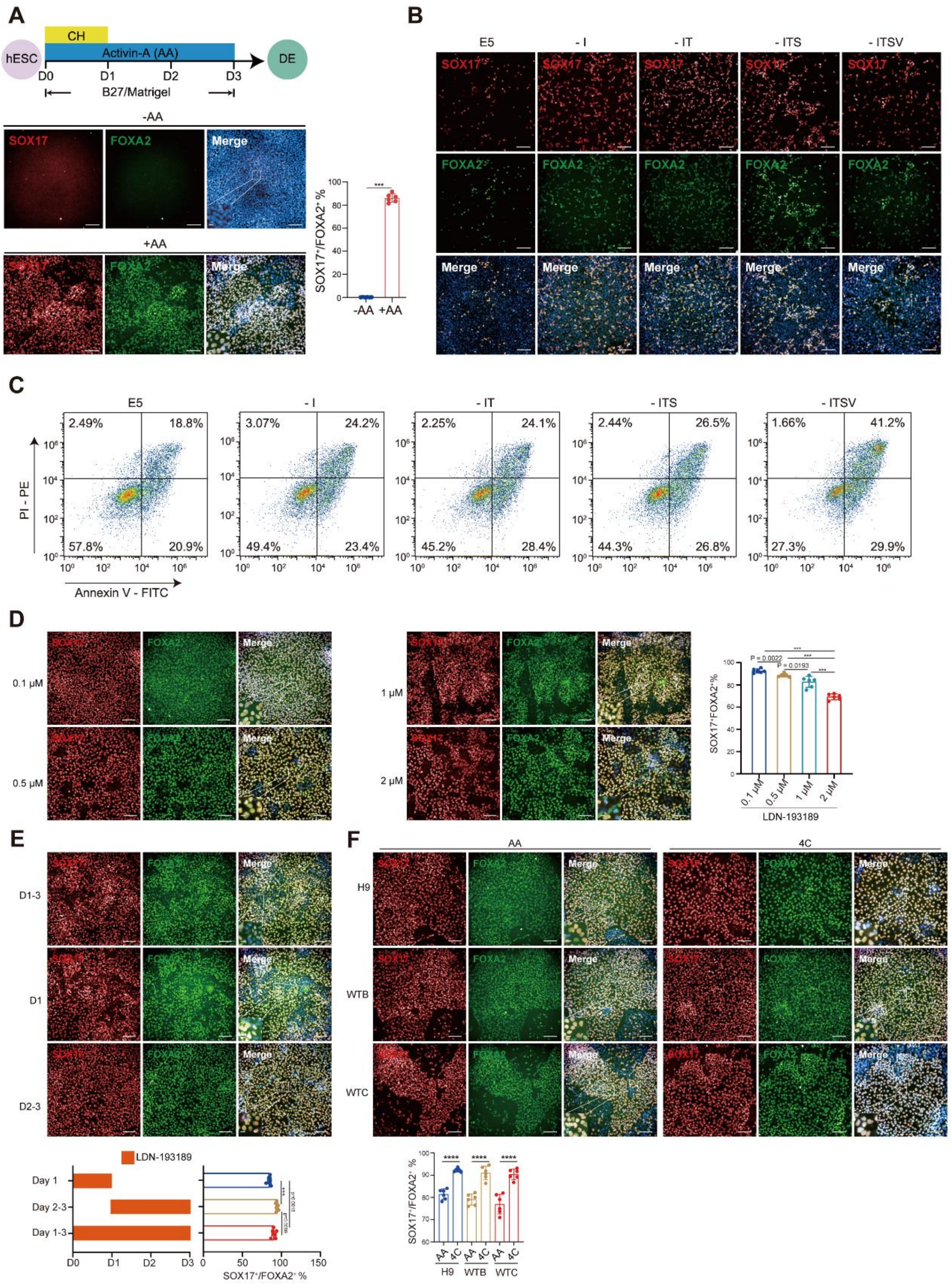

Fig. S1. Optimization of the fully synthetic system for highly efficient DE induction from hPSCs. (A) Immunofluorescence analysis of DE markers SOX17 and FOXA2 on hESCs differentiated with or without AA for 3 days as illustrated by the upper schematic (n = 6 biologically independent experiments). CH, CHIR99021. Scale bars, 50 μm. (B) Immunofluorescence

analysis of SOX17 and FXOA2 of hESCs differentiated under the indicated conditions for 3 days (n = 6 biologically independent experiments). Scale bars, 50 μm. A minus mark demonstrates withdrawal of the indicated component. I, insulin; T, Transferrin; S, sodium selenite; V, Vitamin C. (C) Flow-cytometric analyses of Annexin V and propidium iodide (PI) in conditions in B (n = 6 biologically independent experiments). (D, E) Determination of the optimal concentration (D) and treating window (E) for LDN-193189 in 4C by immunofluorescence analysis of SOX17 and FOXA2 (n = 6 biologically independent experiments). Scale bars, 50 μm. (F) Immunofluorescence analysis of SOX17 and FOXA2 in D3 4C-DE and AA-induced DE from H9 hESCs, as well as the WTB and WTC hiPSC lines (n = 6 biologically independent experiments). Scale bars, 50 μm.

Data are represented as mean ± SE. ***$P < 0.001$; **** $P < 0.0001$.

Fig. S2 (Related to Fig. 3)

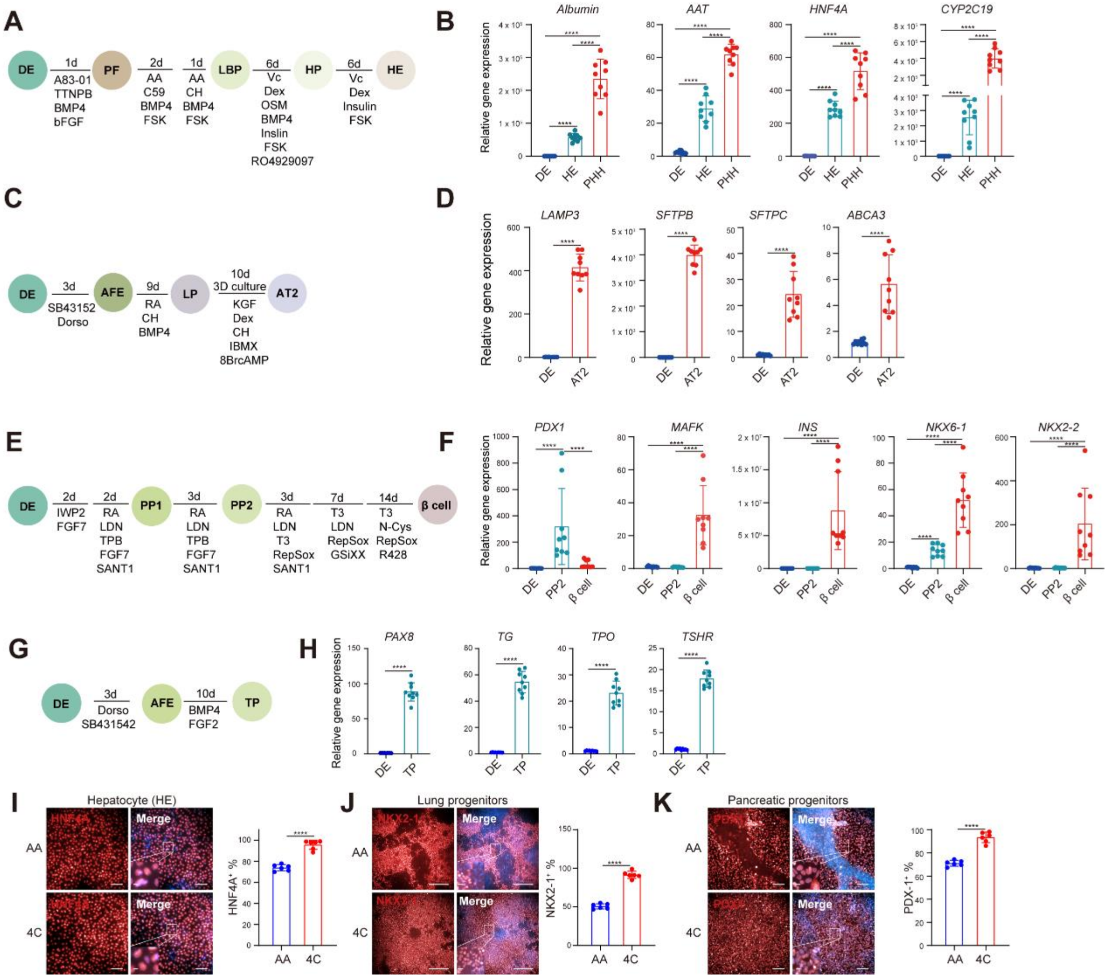

Fig. S2. Multipotent nature of 4C-DE in vitro. (A-H) Protocol outline and qRT-PCR analyses of key marker gene expression in 4C-DE that exposed to hepatic (A and B), lung AT2 (C and D), pancreatic (E and F), and thyroid (G and H) differentiation conditions (n = 9 biologically independent experiments). (I–K). Immunofluorescence analysis of hepatocyte (I), lung progenitor (J), and pancreatic progenitor (K) markers in 4C- or AA-induced DE that exposed to each differentiation condition (n = 6 biologically independent experiments). Scale bars, 50 μm. Data are represented as mean ± SE. **** P < 0.0001.

Fig. S3 (Related to Fig. 3)

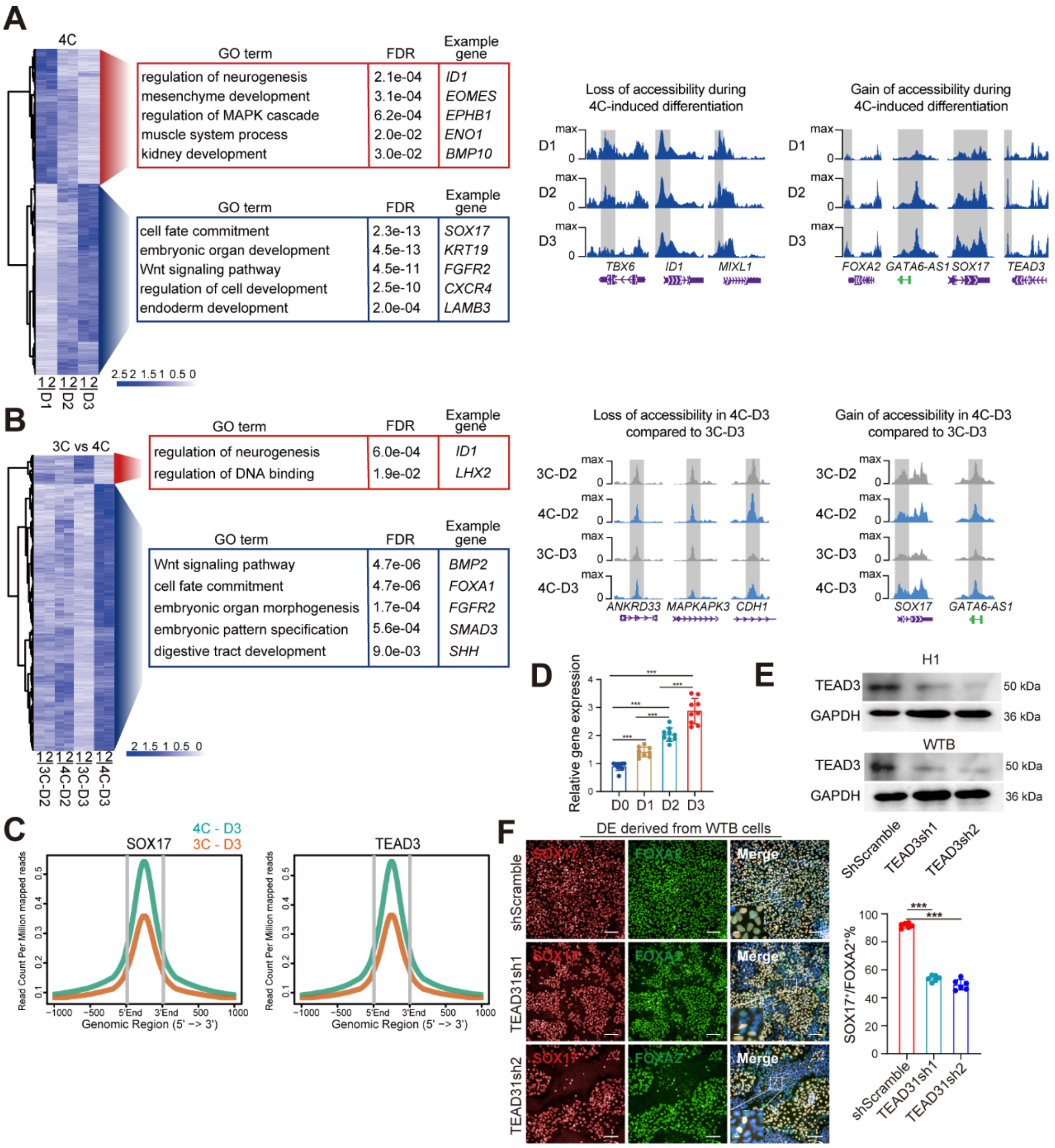

Fig. S3. 4C reconfigures chromatin architecture to specify the DE identify. (A) Chromatin accessibility arranged into groups comparing multiple time points during 4C-induced DE differentiation, with GO analysis of genes in each cluster and an example gene for each GO term. Ring panels display representative loci with reduced or increased chromatin accessibility. (B) Chromatin accessibility arranged into groups comparing the 4C and 3C method, with GO analysis of genes in each cluster. (C) Foot-printing analysis of SOX17 and TEAD3. (D) qRT-PCR analysis of TEAD3 expression during 4C-induced DE specification (n = 9 biologically independent experiments). (E) Western blot analysis of the knockdown efficiency in shScramble control and two TEAD3 knockdown (shTEAD3-1 and shTEAD3-2) hESC (H1, upper panel) or hiPSC (WTB, lower panel) lines differentiated with 4C for 3 days. (F) Immunofluorescence analysis of SOX17 and FOXA2 in shScramble control and two TEAD3 knockdown (shTEAD3-1 and shTEAD3-2) WTB hiPSC lines differentiated with 4C for 3 days (n = 6 biologically independent experiments). Scale bars, 50 μm. Data are represented as mean ± SE. *** P < 0.001; **** P < 0.0001.

Table S1. The positive hits promoting DE differentiation via a high-content imaging and analyzing system

No.	Full Name	Function (s)
1	LDN-193189	BMP inhibitor
2	AG690	PARP-1 inhibitor
3	LY294002	PI3K inhibitor
4	CC-930	JNK inhibitor
5	LDN-212854	BMP receptor inhibitor
6	PIK-90	PI3K inhibitor
7	SB203580	MAPK inhibitor
8	DMH1	BMP receptor inhibitor
9	Rapamycin	mTOR inhibitor
10	AZD8055	mTOR inhibitor
11	Lithium chloride (LiCl)	GSK3 inhibitor
12	LP-922761	AAK1 inhibitor
13	UK-383367	BMP inhibitor
14	LY-411575	γ-secretase inhibitor
15	TWS119	GSK3 inhibitor
16	RKI-1447	ROCK inhibitor
17	JNK-IN-8	JNK inhibitor
18	Nutlin-3	P53 inhibitor
19	SP600125	JNK inhibitor
20	Metformin	MAPK inhibitor
21	SU3327	JNK inhibitor
22	KN-62	CaMKⅡ inhibitor
23	KRIBB11	HSF1 inhibitor
24	CID75563	PKD inhibitor
25	UNC0379	SETD8 inhibitor
26	I-BET151	BRD2 inhibitor
27	FT113	FAS inhibitor
28	AUZ454	CDK2 inhibitor
29	TAPI-1	ADAM17 inhibitor
30	SU11274	MET inhibitor
31	MK-8245	SCD inhibitor
32	Fatostatin	SREBPs inhibitor
33	Orlistat	Lipase inhibitor
34	MF-438	SCD inhibitor
35	A939572	SCD inhibitor
36	MK-8245	SCD inhibitor
37	C75	FASN inhibitor
38	SKF-86002	MAPK inhibitor
39	EPZ004777	DOT1L inhibitor

40	DMH2	BMP inhibitor
41	DAPT	γ-secretase inhibitor
42	A-196	SUV420H1/2 inhibitor
43	Clinofibrate	HMGCR inhibitor
44	SANT1	Smo inhibitor
45	BIX-01294	Histone methyltransferase inhibition
46	C646	P300 inhibitor
47	SGC 0946	DOT1L inhibitor

Table S2. Summary of protocols to generate DE from hPSCs.

**Medium**	**Factors**	**Matrix**	**Time**	**Efficiency**	**Reference**
CDM2	Activin-A<br>CHIR99021<br>PI-103<br>DM3189	Fibronectin<br>or Matrigel	72h	> 90%	[1]
RPMI-1640	B27 Supplement<br>CHIR99021<br>Activin-A	Matrigel	72h	> 76%	[2,3]
Advanced<br>RPMI-1640	Activin-A<br>CHIR99021<br>JNK-IN-8	Vitronectin	72h	> 91%	[4]
RPMI-1640	CHIR99021<br>Dorsomorphin<br>Human serum<br>albumin<br>Vitamin C	iMatrix-511	96h	> 87%	[5]
DMEM/F12	CHIR99021<br>LDN-193189<br>Vitamin C	Vitronectin,<br>laminin-521<br>or Matrigel	72h	> 96%	This study

Table S3. Quantitative RT-PCR primers

Gene	Forward Primer (5' to 3')	Reverse Primer (5' to 3')
SOX17	ACGCCGAGTTGAGCAAGAT	ACTTGTAGTTGGGGTGGTCC
FOXA2	CCACCCGTTCTCCATCAACA	CCACCCGTTCTCCATCAAC
GATA4	AAGACACCAGCAGCTCCTTC	TTGATGGGACGCATCTCCTC
GATA6	GCCAACTGTCACACCACAAC	ATAGCAAGTGGTCTGGGCAC
EOMES	ATTCCACCGCCACCAAACT	TGCCACAGGTCACCCATTT
CXCR4	TGTGGGTGGTTGTGTTCCA	CCCTTGGAGTGTGACAGCTT
ALB	TATGCCCCGGAACTCCTTTT	TGGCACACTTGAGTCTCTGT
AAT	GCATGGGAAGCTGGGAAATT	ATCTGCCGTCATGAGAACCA
HNF4A	TGCCTACCTCAAAGCCATCA	ATCTGCTCGATCATCTGCCA
CYP2C19	GGTCCTTGTGCTCTGTCTCT	CATATCCATGCAGCACCACC
LAMP3	CCTGCAGGTGAAAACAACCG	ATGGCCCCAATCACAGGAAG
SFTPB	GAAGTCTGGGGACATGTGGG	TGTCCTGGAAAATGGCCTCC
SFTPC	GTGAGCAGGGTCAGTGGAAG	CCCATTTCTCCAGGGCTTGT
ABCA3	TCTTCGAGCACCCCTTCAAC	GTAGTGTGCCAGCCTTCTGT
PDX1	GCTGCCTTTCCCATGGATGA	GCTGCCTTTCCCATGGATGA
MAFK	TGACGACTAATCCCAAACCGA	TGGACACCAGCTCATCATCG
INS	TCTACCTAGTGTGCGGGGAA	GTTCCACAATGCCACGCTTC
NKX6-1	TGGCCTATTCGTTGGGGATG	AGTCCTGCTTCTTCTTGGCC
NKX2-2	CTTCTACGACAGCAGCGACA	CGGGGTCTCCTTGTCATTGT
TEAD3	CACCTGTTTGTGCACATCGG	GGCCCAGAACTTGACAAGGA

Supplemental Experimental Procedures

High-throughput chemical screening

High-throughput small molecule screening was carried out using the platform and method we established previously [7,8]. Briefly, H1 hESCs were seeded onto Synthemax-coated 384-well plates at a density of 8,000 cell/ in E8 medium and grown to ~80% confluency. Culture medium was then switched to DMEM/F12 medium supplemented with 71 μg ml^{-1} Vc and 3 μM CHIR99021. In the same time, chemicals at the concentration of 1 μM from in-house-generated library [9] consisting of 735 small molecules were added into the medium by using a Tecan Freedom EVO 150 liquid handler. 24 hours later, CHIR99021 was removed and the culture medium was renewed with DMEM/F12 containing only Vc and chemicals of the library. 100 ng ml^{-1} AA was used as the positive control. Cells were immunostained with a FOXA2 antibody (Cell Signaling Technology, 8186S) at day 3 and images were captured and quantified by the Operetta CLS High-Content Analysis System (PerkinElmer, USA) and the Harmony 4.9 software (PerkinElmer, USA).

Quantitative RT-PCR

Total RNA was isolated from the collected cells using RNAzol (Molecular Research Center, RN190). cDNA was then produced by using a HiScript II 1st Strand cDNA Synthesis Kit (Vazyme, R211-01). qRT-PCR was performed in triplicate using the AceQ Universal SYBR qPCR Master Mix (Vazyme, Q511-02) on the Roche LightCycler 480. All primers used for qRT-

PCR are listed in **Table S3**.

Immunofluorescence staining and quantification

Cells were fixed with 4% paraformaldehyde for 30 minutes at room temperature (RT). After washing with PBS for three times, 5 minutes each, cells were blocked and permeabilized using 0.5% Triton X-100 and 3% bovine serum albumin (BSA, Solarbio, A8010) buffer for 30 minutes at RT. Then cells were stained with antibodies to SOX17 (R&D, AF1924), FOXA2, GATA4 (Santa Cruz, sc-25310), GATA6 (Cell Signalling Technology, 5851), HNF4A (Cell signalling Technology, 3113), SFTPC (Abcam, ab211326), PDX1 (R&D, AF2419), AAT (Abcam, ab166610), NKX2-2 (R&D, MAB8167-SP), and C-peptide (R&D, MAB14171-SP) at 4 ℃ overnight. Secondary antibody staining was performed for 2 hours at RT. DAPI (Sigma, D9542) staining was used to visualize the nucleus. The immunofluorescent images were acquired and quantified using the Operetta CLS High-Content Analysis System, while gene expression quantification was performed utilizing the Harmony 4.9 software.

Cell apoptosis analysis

Cells were dissociated into single cell using Accutase and collected for Annexin V-APC/7-AAD staining. Each cell pellet was resuspended in 500 μl PBS containing 3% BSA. Then, 5 μl Annexin V-APC antibody (BD Biosciences, 550475) and 5 μl 7-AAD (BD Biosciences, 559925) were added to the cells and incubated for 15 minutes at 37℃. The apoptotic cells were then analysed by using the CytoFLEX S Flow Cytometry System (Beckman).

Differentiation of 4C-induced DE (4C-DE) cells

Hepatic differentiation of 4C-DE was performed as previously described [10,11]. Briefly, 4C-DE were cultured in HE differentiation medium I (IMDM (Hyclone, SH30228.01)/ Ham's F12 (Hyclone, SH30026.01) (1:1, vol/vol) consist of 0.1% poly vinyl alcohol (Sigma, 8136), 1% chemically defined lipid concentrate (ThermoFisher, 11905031), and 10% knockout serum replacement (KSR, ThermoFisher, 10828028)) supplemented with 1 μM A83-01 (Sigma, SML0788), 75 nM TTNPB (Selleck, S4627), 30 ng ml^{-1} BMP4 (R&D, AFL314E), and 10 ng ml^{-1} FGF2 (Peprotech, AF-100-18B)) for 1 day. Cells were then cultured in HE differentiation medium I supplemented with 1 μM C59 (Selleck, S7037), 1 μM Forskolin (Selleck, S2449), 10 ng ml^{-1} AA, and 30 ng ml^{-1} BMP4 for 2 days. C59 was then replaced with 1 μM CHIR99021 and cells were cultured for another day. Thereafter, cells were treated with HE differentiation medium II (IMDM/F12 (1:1, vol/vol) consist of 1% chemically defined lipid concentrate and 15 μg ml^{-1} transferrin (Sigma, T0665)) supplemented with 2 μM Ro4929097 (Selleck, S1575), 10 μM Dexamethasone (Selleck, S1322), 10 μM Forskolin, 10 μg ml^{-1} BMP4, 10 ng ml^{-1} Oncostatin M (OSM, R&D, 295-OM), 200 μg ml^{-1} Vc, and 10 μg ml^{-1} insulin (Sigma, 91077C) for 6 days. Finally, cells were cultured in HE differentiation medium II supplemented with 2 μM Ro4929097, 10 μM Dexamethasone, 10 μM Forskolin, 200 μg ml^{-1} Vc, and 10 μg ml^{-1} insulin for 6 days.

Differentiation of 4C-DE into lung alveolar cells was performed according to previously studies [12,13]. Briefly, 4C-DE were differentiated with the complete serum-free differentiation medium (cSFDM) containing IMDM/F12 (3:1, vol/vol), 1% B27 supplement (ThermoFisher, 17504044), 0.5% N2 Supplement (ThermoFisher, 17502001), 0.05% BSA, 4.5×10^{-4} M monothioglycerol (Sigma, M6145), 1×Glutamax (ThermoFisher, 35050061), and 50 μg ml^{-1} Vc. Differentiation was initiated by dissociating the 4C-DE cells with 0.5 mM EDTA for 4 minutes at RT and passaged at a ratio between 1:2 to 1:6 into 12-well matrigel-coated plates in cSFDM supplemented with 10 μM SB431542 (Selleck, S1067) and 2 μM Dorsomorphin (Selleck,

S7840). During the first 24 hours after passaging, 10 μM Y-27632 was added to the medium to enhance cell survival. 72 hours after, medium was changed to cSFDM supplemented with 3 μM CHIR99021, 10 ng ml^{-1} BMP4 and 100 nM retinoic acid (Selleck, S1653) for 9 days. Then cells were dissociated using 0.05% Trypsin and washed in FBS-containing medium. Cells were pelleted by centrifugation at 200g for 5 min and resuspended in matrigel in droplets of 20-50 μl at a density of 1 × 10^3 cells/μl. Subsequently, cells were plated on a pre-warmed tissues culture plate. After allowing the droplets to solidify for 20 min in a 37℃ incubator, pre-warmed cSFDM supplemented with 3 μM CHIR99021, 10 ng ml^{-1} KGF (Peprotech, 100-19), 50 nM Dexamethasone, 0.1 mM 8BrcAMP (Selleck, S7857), 0.1 mM IBMX (Sigma, I5879) and 10 μM Y-27632 was added. After 3-4 days, fresh medium was added without Y-27632, and with further medium replacement performed every 4-5 days, Transmission electron microscopy analysis of the alveolospheres was performed according to a standard method as previously described [12].

Pancreatic differentiation of 4C-DE was carried out using an established protocol [14,15], which is briefly outlined as follows: **Step 1**: 4C-DE cells were cultured in MCDB131 medium (ThermoFisher, 21875059) supplemented with 0.5% BSA, 50 ng ml^{-1} FGF7 (SinoBiological, 10210-H07E), 0.25 mM Vc, 1.25 μM IWP2 (S7085, Selleck) for 2 days. **Step 2**: cells were treated with MCDB131 medium supplemented with 2% BSA, 0.25 mM Vc, 50 ng ml^{-1} FGF7, 0.25 μM SANT-1 (TargetMol, T2450), 1 μM retinoic acid, 0.1 μM LDN193189, 1×ITS-X (ThermoFisher, 51500056), and 0.2 μM TPB (Millipore, 565740) for 2 days. **Step 3**: cells were cultured in MCDB131 medium supplemented with 10 mM glucose (Sigma, G6152), 2% BSA, 0.25 μM SANT-1, 0.05 μM retinoic acid, 0.1 μM LDN193189, 1×ITS-X, 1 μM T3 (TargetMol, T1669), 10 μM RepSox (TargetMol, T6337), 10 μM zinc sulfate (Sigma, Z0251), and 10 μg ml^{-1} heparin (Selleck, S1346) for 3 days. **Step 4**: cells were incubated with MCDB131 medium supplemented with 20 mM glucose, 2% BSA, 0.1 μM LDN193189, 1×ITS-X, 1 μM T3, 10 μM RepSox, 10 μM zinc sulfate, and 0.1 μM gamma secretase inhibitor XX (TargetMol, T6135) for 7 days. **Step 5**: cells were cultured MCDB131 medium supplemented with 2% BSA, 1×ITS-X, 1 μM T3, 10 μM RepSox, 10 μM zinc sulfate, 1 mM N-acetyl cysteine (Sigma, A9165), 10 μM Trolox (Millipore, 648471), 2 μM R428 (Selleck, S2841), and 10 μg ml^{-1} heparin for 15 days.

Differentiation of 4C-DE into thyroid progenitors was performed according to previously studies [16]. Briefly, 4C-DE were differentiated with the complete cSFDM. Differentiation was initiated by dissociating the 4C-DE cells with 0.5 mM EDTA for 4 minutes at RT and passaged at a ratio between 1:2 to 1:6 into 12-well matrigel-coated plates in cSFDM supplemented with 10 μM SB431542 and 2 μM Dorsomorphin. During the first 24 hours after passaging, 10 μM Y-27632 was added to the medium to enhance cell survival. 72 hours after, medium was changed to cSFDM supplemented with 100 ng ml^{-1} FGF2 and100 ng ml^{-1} BMP4 for 10 days.

Functional assays of 4C-DE-derived hepatocytes

Periodic acid Schiff (PAS) staining analysis of the 4C-DE-derived hepatocytes was performed using a PAS Staining Kit (Solarbio, G1280) according to the manufacturer's instruction. Uptake of acetylated low-density lipoprotein (acLDL) was assessed by incubating cells with 4 μg/ml of ac-LDL conjugated with Alexa Fluor-594 (ThermoFisher, L35353) for 30 minutes at 37℃. After incubation, cells were washed with PBS and fixed with 4% formaldehyde before examination with fluorescence microscopy. For indocyanine green (ICG) uptake assays, ICG (MCE, HY-D0711) was dissolved in DMSO at 5 mg ml^{-1} and freshly diluted to 1 mg ml^{-1} with the cell culture medium. Cells were then incubated with the diluted ICG for 30 minutes at 37℃, washed with

the PBS, and analysed by using a Leica DMi8 inverted microscope. Albumin secretion of the 4C-DE-derived hepatocytes was detected by using a Human Albumin ELISA Kit (Abclonal, RK00157) according to the manufacturer's instruction.

Differentiation analysis of 4C-DE *in vivo*

4C-DE were dissociated with Accutase, centrifuged at 200g for 3 minutes, and resuspended in 0.1 ml matrigel. 5×10^6 cells were injected subcutaneously into the groin of the 8-week-old NOD-SCID mice (GemPharmatech). Nine weeks after injection, resultant matrigel plugs/transplants were surgically removed from the mice, fixed with 4% paraformaldehyde, embedded in paraffin, and analysed by haematoxylin-eosin staining or immunohistochemistry staining using antibodies against HNF4A (Cell signalling Technology, 3113), AAT (Abcam, ab166610), cTNT (ThermoFisher, MA5-12960), AFP (R&D, MAB1368), αSMA (ThermoFisher, 19245), and choline acetyltransferase (CHAT) (Abcam, ab181023). Slides were imaged with an Upright metallurgical microscope (Olympus BX51). Animal experiments were approved by the institutional ethics and animal welfare committee of Sun Yat-sen University.

Lentivirus production and gene knockdown (KD) experiments

shRNAs for knockdown of *TEAD3* (target sequence 1: 5'-GCCACTGTTCTGCGCTTTAAT-3' and target sequence 2: 5'-CCATGTCTACAAGCTCGTCAA-3') or a scramble control (target sequence: 5'-CCTAAGGTTAAGTCGCCCTCG-3') were selected from a shRNA library predesigned by Sigma (http://www.sigmaaldrich.com/life-science/functional-genomics-and-rnai/sirna/mission-predesigned-sirna.html). The shRNA primers were subcloned into the pLKO.1-blast (Addgene, 26655) vector and confirmed by Sanger sequencing. KD lentivirus were made by co-transfection of HEK293T cells with the Lentiviral pLKO.1 shRNA-expressing vector, an envelope plasmid (pMD2.G, Addgene, 12259), and a packaging plasmid (psPAX2, Addgene, 12260) using Lipofectamine™ 2000 (ThermoFisher, 11668019). Then virus-containing medium was collected from the HEK293T cells at 24 and 48 hours post-transfection and filtered through a 0.45 μm filter. H1 hESCs were subjected to two rounds of viral infection (6 hours per round) with the presence of 8 μg ml^{-1} polybrene. 48 hours after the last infection, transduced cells were selected with 10 μg ml^{-1} blasticidin (Selleck, S7419) for 3 continuous passages. KD efficiencies of the targeted genes were evaluated by RT-qPCR.

Severe acute respiratory syndrome coronavirus 2 (SARS-CoV-2) entry virus construction, production, and transfection

Pseudoviruses of SARS-CoV-2 were generated through co-transfection of 293T cells with psPAX2, pCDH-EF1α-MCS-BGH-PGK-GFP-T2A-Puro (SBI, CD550A-1), and pCMV3-SARS-CoV-2 Spike (SinoBiological, VG40799-UT) using the jetPRIME transfection reagent (Polyplus, 114-15). The supernatants were collected at 24 and 48 hours post-transfection and filtered through a 0.45 μm filter. Lung alveolar organoids were dissociated using TrypLE Express Enzyme (ThermoFisher, 12605028) and incubated with the pseudovirius at 37 ℃ for 2 hours. Transfected cells were then washed with PBS, resuspended in ice-cold matrigel, and plated as a drop of 20 μl per well in a 24-well plate. Plates were placed in an incubator (37 ℃, 5% CO_2) for 20 minutes to allow the matrigel to solidify before adding the cell culture medium. Reaggregated cell plus were analyzed for GFP expression 24 hours later.

RNA sequencing (RNA-seq) and data analysis

Total RNA of cells was isolated using RNAzol. Sequencing libraries were constructed using the VAHTS Universal V8 RNA-seq Library Prep Kit for Illumina (Vazyme, NR605-1) according

to the manufacturer's instruction. For RNA-seq analysis, sequence quality was assessed using FastQC and the raw RNA-seq reads were aligned to the human genome (hg19) by Hisat2 (version 2.1.0). Alignment files were sorted by SAMtools (version 1.9). HTSeq (version 0.11.2) was used to assign reads to genes and summarized as gene-level counts. DESeq2 package (version 1.30.1) in R was used to analyze gene differential expression by comparing transcriptomes of each sample using a criterial of adjusted *P*-value<0.05, a log2 fold change>1. The differential expression heatmap was produced using the R package pheatmap. Gene ontology enrichment analysis and KEGG enrichment analysis were performed using the R package clusterProfiler.

Assay for transposase-accessible chromatin sequencing (ATAC-seq) and data analysis

ATAC-seq sequencing libraries were constructed using the TruePrep DNA Library Prep Kit V2 for Illumina (Vazyme, TD501) according to the manufacturer's instruction. Quality control of the libraries was performed using an Agilent 2100 Bioanalyzer and sequencing was performed using an Illumina NovaSeq 6000 Sequencer.

For ATAC-seq analysis, raw sequencing reads were mapped to human genome (hg19) by Bowtie2 (version 2.3.5). PCR duplicates were removed by Picard (version 1.124) and the mitochondrial DNA reads were removed by awk. ATAC-seq peaks were called using MACS2 (version 2.2.5). Peaks from the biological replicates were merged using SAMtools and Bedtools. The ATAC-seq data were visualized by using the WashU Epigenome Browser. Raw counts in peaks were normalized and the specific peaks were identified with a criterial of adjusted *P*-value<0.05, a log2 fold change>1 using the DESeq2.

Supplemental References

1. Loh, K.M., Ang, L.T., Zhang, J., Kumar, V., Ang, J., Auyeong, J.Q., Lee, K.L., Choo, S.H., Lim, C.Y., Nichane, M., et al. (2014). Efficient endoderm induction from human pluripotent stem cells by logically directing signals controlling lineage bifurcations. Cell Stem Cell *14*, 237-252. 10.1016/j.stem.2013.12.007.

2. Li, Q., Hutchins, A.P., Chen, Y., Li, S., Shan, Y., Liao, B., Zheng, D., Shi, X., Li, Y., Chan, W.Y., et al. (2017). A sequential EMT-MET mechanism drives the differentiation of human embryonic stem cells towards hepatocytes. Nat Commun *8*, 15166. 10.1038/ncomms15166.

3. Hogrebe, N.J., Augsornworawat, P., Maxwell, K.G., Velazco-Cruz, L., and Millman, J.R. (2020). Targeting the cytoskeleton to direct pancreatic differentiation of human pluripotent stem cells. Nat Biotechnol *38*, 460-470. 10.1038/s41587-020-0430-6.

4. Li, Q.V., Dixon, G., Verma, N., Rosen, B.P., Gordillo, M., Luo, R., Xu, C., Wang, Q., Soh, C.L., Yang, D., et al. (2019). Genome-scale screens identify JNK-JUN signaling as a barrier for pluripotency exit and endoderm differentiation. Nat Genet *51*, 999-1010. 10.1038/s41588-019-0408-9.

5. Jiang, Y., Chen, C., Randolph, L.N., Ye, S., Zhang, X., Bao, X., and Lian, X.L. (2021). Generation of pancreatic progenitors from human pluripotent stem cells by small molecules. Stem Cell Reports. 10.1016/j.stemcr.2021.07.021.

6. Guo, D., Liu, H., Ruzi, A., Gao, G., Nasir, A., Liu, Y., Yang, F., Wu, F., Xu, G., and Li, Y.X. (2017). Modeling Congenital Hyperinsulinism with ABCC8-Deficient Human Embryonic Stem Cells Generated by CRISPR/Cas9. Sci Rep *7*, 3156. 10.1038/s41598-017-03349-w.

7. Ji, X., Meng, Y., Wang, Q., Tong, T., Liu, Z., Lin, J., Li, B., Wei, Y., You, X., Lei, Y., et al.

(2023). Cysteine-Based Redox-Responsive Nanoparticles for Fibroblast-Targeted Drug Delivery in the Treatment of Myocardial Infarction. ACS Nano *17*, 5421-5434. 10.1021/acsnano.2c10042.

8. Xu, H., Liu, G., Gong, J., Zhang, Y., Gu, S., Wan, Z., Yang, P., Nie, Y., Wang, Y., Huang, Z.P., et al. (2022). Investigating and Resolving Cardiotoxicity Induced by COVID-19 Treatments using Human Pluripotent Stem Cell-Derived Cardiomyocytes and Engineered Heart Tissues. Adv Sci (Weinh) *9*, e2203388. 10.1002/advs.202203388.

9. Wang, J., Gu, S., Liu, F., Chen, Z., Xu, H., Liu, Z., Cheng, W., Wu, L., Xu, T., Chen, Z., et al. (2022). Reprogramming of fibroblasts into expandable cardiovascular progenitor cells via small molecules in xeno-free conditions. Nat Biomed Eng *6*, 403-420. 10.1038/s41551-022-00865-7.

10. Ang, L.T., Tan, A.K.Y., Autio, M.I., Goh, S.H., Choo, S.H., Lee, K.L., Tan, J., Pan, B., Lee, J.J.H., Lum, J.J., et al. (2018). A Roadmap for Human Liver Differentiation from Pluripotent Stem Cells. Cell Rep *22*, 2190-2205. 10.1016/j.celrep.2018.01.087.

11. Loh, K.M., Palaria, A., and Ang, L.T. (2019). Efficient Differentiation of Human Pluripotent Stem Cells into Liver Cells. J Vis Exp. 10.3791/58975.

12. Jacob, A., Morley, M., Hawkins, F., McCauley, K.B., Jean, J.C., Heins, H., Na, C.L., Weaver, T.E., Vedaie, M., Hurley, K., et al. (2017). Differentiation of Human Pluripotent Stem Cells into Functional Lung Alveolar Epithelial Cells. Cell Stem Cell *21*, 472-488 e410. 10.1016/j.stem.2017.08.014.

13. Jacob, A., Vedaie, M., Roberts, D.A., Thomas, D.C., Villacorta-Martin, C., Alysandratos, K.D., Hawkins, F., and Kotton, D.N. (2019). Derivation of self-renewing lung alveolar epithelial type II cells from human pluripotent stem cells. Nat Protoc *14*, 3303-3332. 10.1038/s41596-019-0220-0.

14. Rezania, A., Bruin, J.E., Arora, P., Rubin, A., Batushansky, I., Asadi, A., O'Dwyer, S., Quiskamp, N., Mojibian, M., Albrecht, T., et al. (2014). Reversal of diabetes with insulin-producing cells derived in vitro from human pluripotent stem cells. Nat Biotechnol *32*, 1121-1133. 10.1038/nbt.3033.

15. Mahaddalkar, P.U., Scheibner, K., Pfluger, S., Ansarullah, Sterr, M., Beckenbauer, J., Irmler, M., Beckers, J., Knobel, S., and Lickert, H. (2020). Generation of pancreatic beta cells from CD177(+) anterior definitive endoderm. Nat Biotechnol *38*, 1061-1072. 10.1038/s41587-020-0492-5.

16. Kurmann, A.A., Serra, M., Hawkins, F., Rankin, S.A., Mori, M., Astapova, I., Ullas, S., Lin, S., Bilodeau, M., Rossant, J., et al. (2015). Regeneration of Thyroid Function by Transplantation of Differentiated Pluripotent Stem Cells. Cell Stem Cell *17*, 527-542. 10.1016/j.stem.2015.09.004.
